# On the Induction of Abortion as a Therapeutic Measure

**Published:** 1882-02

**Authors:** 


### ON THE INDUCTION OF ABORTION AS A THERAPEUTIC MEASURE.

Dr. Priestley read a paper before the Obstetrical Society of London on the above subject. The author considered that the indications for the induction of abortion as distinct from the induction of premature labor had never been laid down with sufficient precision in this country (*Med. Times and Gazette*). It was usual to say that each case must be judged on its merits, and this lack of rules might unfortunately lead to serious abuse. Examples had repeatedly come within his knowledge where abortion had been provoked for reasons which seemed to him quite inadequate. Though the medical man was no doubt acting in good faith in these cases, it would have been very difficult to sustain his action in a court of law. For instance, in one case abortion was induced at the fourth or fifth month on account of a bad rupture of the perineum at the last confinement. In a succeeding pregnancy a sound was introduced with a similar object at the end of a month. This, however, had no effect, and she went the full term, and had an easy and natural labor. In a second instance an attempt was made to induce abortion at the second month because the patient had aborted not long before, and it was feared that pregnancy had recurred too speedily, while a much-desired journey would have to be postponed if miscarriage recurred at the same period as before. Fortunately the attempt failed, and the patient went to her full term. It was often necessary to remind wives and mothers that even spontaneous abortion is sometimes more damaging to health than natural parturition, more frequently lays the foundation of disease, and, if repeated, abridges the period of youth and comeliness. These risks were necessarily greater if abortion was induced. The reasons which may be adduced as justifying the induction of abortion are the following:

1. Pelvic deformity so great as to preclude the birth of a viable child.

2. Narrowing of the genital canal by tumors, cicatrices, or cancer, so as to prevent the passage of a viable child. Great care was here necessary to not over-estimate the amount of obstruction. If a series of cases of Cæsarean section with fair success should occur, the reasons for inducing abortion in such instances would be undermined. In cases of cancer there was fair ground for this operation, since the woman had but a short time to live in any case.

3. In obstinate vomiting of pregnancy, when all other expedients are fruitless, and a fatal result is anticipated if relief can not be afforded.

4. In eclampsia, abortion should only be induced as a last resort to save life.

5. In irreducible retroversion or retroflexion of the gravid uterus, but only when life is seriously threatened, not merely because the displacement is irreducible.

6. In severe hemorrhage.

7. In certain other diseases where the complication of pregnancy is undoubtedly endangering life.

The responsibility of inducing abortion should never be undertaken without a consultation of two or more medical men, and M. Tarnier had even suggested that a legal declaration should be made to the public prosecutor in every case, he would lay it down that the induction of abortion is only legitimate when the life of the mother is so imperiled by the continuance of pregnancy that emptying the uterus presents itself as the only alternative to save the mother. In insanity, chorea, and the like, the proper treatment was probably to treat the morbid conditions, and leave the pregnancy to take care of itself.—*Obstetric Gazette.*

